# Pharmacogenomics: The Genomics of Drug Response

**DOI:** 10.1002/(SICI)1097-0061(200004)17:1<16::AID-YEA6>3.0.CO;2-E

**Published:** 2000

**Authors:** Ruth March

**Affiliations:** Research & Development Genetics AstraZeneca, Mereside Alderley Park, Macclesfield Cheshire SK10 4TG UK

## Abstract

Pharmacogenomics is defined as the study of the association between genetics and drug response. This is a rapidly expanding field with the hope that, within a few years, prospective genotyping will lead to patients being prescribed drugs which are both safer and more effective (‘the right drug for the right patient’, or personalized medicine). There are many existing examples in the literature of strong associations between genetic variation and drug response, and some of these even form the basis of accepted clinical tests. The molecular basis for some of these associations is described, and includes examples of variation in genes responsible for absorption and metabolism of the drug, and in target and disease genes. However, there are many issues surrounding the legal, regulatory and ethical framework to these studies that remain unanswered, and a huge amount of education both for the public and haelthcare professionals will be needed bafore the results of this new madicine can be widely accepted.


**Yeast**
*Yeast* 2000; **17**: 16–21.

**Review Article**

### Pharmacogenomics: the genomics of drug response

Ruth March*
*Research & Development Genetics, AstraZeneca, Mereside, Alderley Park, Macclesfield, Cheshire SK10 4TG, UK*

*Correspondence to:
R. March, Research & Development Genetics, AstraZeneca, Mereside, Alderley Park, Macclesfield, Cheshire SK10 4TG, UK.
E-mail: Ruth.march@astrazeneca.com

Received: 4 February 2000
Accepted: 11 February 2000

### Abstract

**Pharmacogenomics is defined as the study of the association between genetics and drug response. This is a rapidly expanding field with the hope that, within a few years, prospective genotyping will lead to patients being prescribed drugs which are both safer and more effective ('the right drug for the right patient', or personalized medicine). There are many existing examples in the literature of strong associations between genetic variation and drug response, and some of these even form the basis of accepted clinical tests. The molecular basis for some of these associations is described, and includes examples of variation in genes responsible for absorption and metabolism of the drug, and in target and disease genes. However, there are many issues surrounding the legal, regulatory and ethical framework to these studies that remain unanswered, and a huge amount of education both for the public and healthcare professionals will be needed before the results of this new medicine can be widely accepted. Copyright © 2000 John Wiley & Sons, Ltd.**

**Keywords: pharmacogenomics; pharmacogenetics; toxicogenomics; drug response; adverse events; personalized medicine**

### Introduction

During the last months of the twentieth century, a remarkable collaboration was announced. Ten pharmaceutical companies, normally fierce competitors, came together with leading genome research institutions and the Wellcome Trust, to discover, map and place within the public domain 300 000 single nucleotide polymorphisms (SNPs) [6,13,25] across the human genome within 2 years. This $45 million initiative is a result of a new emphasis within the industry on genomics, which is used both to identify susceptibility genes on which new drugs can act, and also to predict which patients will respond to which drugs (personalized medicine, or pharmacogenomics) [9,23,24,32].

In recent years, genomics has provided an exciting and complementary approach to the basic biological research carried out by pharmaceutical researchers and their collaborators to identify genes, or 'targets', for new drugs. The advent of whole-genome screens to map susceptibility genes for multifactorial diseases has meant that pharmaceutical researchers are able to identify even novel genes as drug targets. Successful identification of novel disease genes using this approach was announced in 1999 by Glaxo Wellcome (see **http://www.glaxowellcome.co.uk**). The extension of this approach will depend on the development of high throughput SNP genotyping technology and, more importantly, on the availability of a genome-wide map of SNP markers. The availability of at least 90% of the entire human gene sequence during 2000 (see **http://www.nhgri.nih.gov/NEWS/news.html**), plus the mapping of the SNPs across the genome, should greatly expedite this process.

Another approach which has proved very popular for the identification of novel targets is the use of cDNA microarrays to study gene expression in disease or, more usually, disease models. Genes or sequences whose regulation is altered in disease, as compared to normal tissue, can be identified by this method, and hundreds of gene sequences may be highlighted. Bioinformatics and more conventional genetic studies can be used to prioritize findings from these transcript profiling experiments (see

Copyright © 2000 John Wiley & Sons, Ltd.

**http://www.incyte.com/news/2000/cvt.html** for a description of how this technology was used to identify a new target for cardiovascular disease).

### The genetics of drug response

In contrast to the use of genomics and genetics for the discovery of new drug targets, the use of pharmacogenomics is relatively new in the pharmaceutical industry. This is in spite of the fact that the related science of pharmacogenetics, which traditionally encompassed genetic variation in the enzymes that are responsible for the metabolism of many commonly prescribed drugs, has been studied since the 1950s [26,28,40]. The term 'pharmacogenomics' is generally used to emphasize more recent approaches, such as correlation of genotype with drug response, which could ultimately lead to genotyping individuals before prescription (so-called 'tailored' or 'personalized' medicine), and the use of blood samples taken from individuals taking part in clinical trials for a drug to discover novel genes involved in the disease area under investigation, or in the response of patients to the drug [9,15,23,24,32,34,37] (see **http://www.biospace.com/articles/081999.cfm**). Most large pharmaceutical companies now have pharmacogenetic specialists who are working on the logistics of large-scale genotyping within clinical trials, although data from such studies has yet to be published.

The aim of pharmacogenomics, from the pharmaceutical company's perspective, may include the discovery of new targets from clinical trials, increasing efficiency in the drug discovery process by screening targets for variation, the reduction of costs and timelines in clinical trials by including or excluding certain patients by genotype, the exclusion of patients likely to develop adverse events (side effects) from drug treatment, the differentiation of their product from others in the marketplace (e.g. by identifying patients by genotype who will respond to product X and not to product Y), or simply the testing out of scientific hypotheses about the mode of action of their drug [18].

The aim of pharmacogenomics, from the patient's point of view, is to increase the chance that a prescribed drug will actually alleviate disease, and reduce the incidence of dangerous side adverse drug reactions (ADRs). Even drugs which are highly effective in most individuals typically demonstrate some interindividual variation, so that many patients (between 20% and 75% of the population in clinical trials of 14 major drug categories) derived no clinical benefit from the treatment [38]. A recent survey claimed that ADRs cause more than 100 000 deaths per year, although only a small number of these will be for genetic causes [20].

### The genetics of drug metabolizing enzymes

The first widespread recognition of the importance of pharmacogenetic variation may be traced back to the Second World War, when doctors observed that African–American soldiers given antimalarial drugs were more likely than their Caucasian colleagues to develop haemolytic anaemia. In 1956, the basis for this phenomenon was discovered [5]. Glucose-6-phosphate dehydrogenase is essential for reduction reactions which maintain the integrity of red blood cells. Individuals with mutations in the G6PD gene, leading to G6PD deficiency (which occurs in approximately 10% of African–Americans), are at greatly increased risk of developing haemolytic anaemia when given oxidant drugs such as primaquine and other antimalarial drugs.

Other early examples of pharmacogenetics were seen in response to drugs such as succinylcholine (used to relax muscles during surgery) and the anti-tuberculosis drug isoniazid. It was discovered that about 0.04% of apparently normal individuals were so sensitive to succinylcholine that they had prolonged paralysis of the respiratory muscles after surgery, often needing to be put on a ventilator to maintain breathing. These individuals were found to be homozygous for a polymorphism in the gene for plasma pseudocholinesterase, *CH1* [17], which normally inactivates succinylcholine. Reactions to isoniazid were first noticed in the 1940s, when many individuals developed peripheral neuropathy [16]. This was found to be a result of polymorphisms in the variant *N*-acetyltransferase gene (NAT2), which inactivates isoniazid [8]. There are at least 10 different genotypes at the molecular level resulting in the 'fast' and 'slow' inactivator phenotypes, and they affect reactions to many therapeutic drugs and may also be associated with susceptibility to different forms of cancer [8].

The pharmacogenetic polymorphisms of greatest clinical importance have probably been that of

Copyright © 2000 John Wiley & Sons, Ltd.

the cytochrome P450 genes, particularly that of CYP2D6. This polymorphism was discovered when Bob Smith, of Imperial College, London, took some of the adrenergic-blocking drug debrisoquine which was going through clinical trials in England for the treatment of hypertension. He collapsed with vascular hypotension, caused by an almost complete absence of the normal metabolism of the drug. Mahgoub *et al.* [22] showed that between 3% and 10% of the Caucasian population were so-called 'poor metabolizers' (PMs), and suggested that these individuals were homozygous for a recessive gene (the frequency of the PM phenotype is lower in other ethnic groups; approximately 5% in African–Americans and 1–2% in Asians). It was later found that the 'poor metabolizer' phenotype also affects the metabolism of more than 30 drugs and environmental chemicals, including as much as 20% of all commonly prescribed drugs, probably including codeine [11]. The CYP2D6 gene also has alleles leading to a 'enhanced metabolizer' phenotype, which has been correlated with increased susceptibility to cancer of the bladder, liver, pharynx and stomach, and lung cancer caused by cigarette smoke. Other genes of the large and highly polymorphic P450 family, such as CYP3A4, are involved in the metabolism of many other drugs [9,28,29].

An everyday example of the use of pharmacogenetics in the clinic is the use of the anti-leukaemia drug 6-mercaptopurine. Most individuals metabolize the drug quickly through the action of the drug-metabolising enzyme thiopurine methyl transferase (TPMT). Doses are adjusted to be high enough to treat the leukaemia and prevent relapses. Other individuals metabolize the drug more slowly and need lower doses to avoid the toxic side effects of the drug. A small proportion of individuals metabolize the drug so poorly that its effects can be fatal. These differences are caused by genetic variation (caused by at least four alleles) in TPMT and, after a blood test, individuals are given doses that are tailored to their genetic profile [21,41].

### Pharmacogenetics of target genes

There are many examples where the drug target itself has been shown to be polymorphic, and this can be shown to be associated with variation in drug response both *in vitro* and *in vivo*. The $\mu$ opioid receptor is the primary site of action for most pain-relieving opiate drugs. Bond *et al.* [2] identified a SNP at position 118 of the $\mu$ opioid receptor gene, which was present in approximately 10% of their study population (although there was variation in frequency between ethnic groups). The variant protein was three times more potent in its interaction with $\beta$-endorphin, an endogenous opioid, in test systems than the wild-type protein. Thus, this and other SNPs within the regulatory region of the gene could be linked to phenotypes such as pain perception and stress, and also multifactorial conditions such as drug addiction.

Angiotensin-converting enzyme (ACE) inhibitors are used to reduce blood pressure and proteinuria in patients with hypertension. Patients with a deletion genotype at the intron 16 of the ACE gene have been shown to have higher activity of circulating ACE when compared to patients with the insertion genotype [12]. In a small study, it was found that patients carrying the insertion allele had a significant response to a 6-month course of therapy with enalapril, whereas patients homozygous for the deletion allele had no benefit.

Asthma is a multifactorial condition, and treatments that modify the 5-lipoxygenase pathway are aimed at those patients where leukotrienes contribute to susceptibility to disease. Hence, patients who fail to respond to treatment with ALOX5-pathway modifiers are thought to have asthma that is not dependent on leukotrienes. Silverman *et al.* [35] defined polymorphisms in the promoter region of the ALOX5 gene which were shown to be associated with lower transcription of a reporter gene *in vitro*. Drazen *et al.* [7] then showed that patients with these sequence variants had diminished gene transcription, and therefore decreased ALOX5 product production and a diminished clinical response to treatment with a drug targeting this pathway. Such an effect indicates an interaction between gene promoter sequence variants and drug-treatment responses, i.e. a pharmacogenetic effect of a promoter sequence on treatment responses.

The 2-adrenergic receptor (2AR) agonists are the most widely used agents in the treatment of asthma, and several polymorphisms have been described within the target genes, particularly the $\beta_2$-adrenoreceptor gene. Several studies have shown associations between SNPs in these genes and response to therapy. One study found that homozygotes for one

Copyright © 2000 John Wiley & Sons, Ltd. 

allele were up to 5.3 times more likely to respond to albuterol than homozygotes for the other allele [4].

Serotonin (5-hydroxytryptamine; 5-HT) is a neurotransmitter that is important in many physiological processes. The serotonin transporter (5-HTT) is used as a target for diseases affected by these processes, such as severe depression. A polymorphism within the promoter region of the 5-HTT gene (short or long form) was shown to lead to different transcriptional activity of the gene *in vitro*. Smeraldi *et al.* [36] demonstrated that carriers of the long form of the gene showed a better response to fluvoxamine (and/or augmentation with pindolol, which is used as an augmentation therapy for non-responders to fluvoxamine) than homozygotes for the short variant.

Other therapeutic agents in psychiatric medicine are targeted to 5-HT receptors, of which there are 14 sub-types, many of which are polymorphic. A single amino acid change in the 5HT-1B receptor at position 124 increases the affinity of sumatriptan (used for migraine therapy) for the mutant receptor by more than two-fold. A side effect of this drug, causing coronary vasospasm in a small number of patients, may be partly caused by the high affinity for the vasoconstriction-mediating h5-HT1B receptor in the coronary artery [3]. Clozapine is an atypical antipsychotic used in the treatment of schizophrenia. Arranz *et al.* [1] genotyped a novel -1438-G/A polymorphism detected in the promoter region, and a polymorphism in the coding region of the 5-$HT_{2A}$ receptor gene, in two independent samples of clozapine-treated patients, including responders and non-responders. A combined analysis of both samples showed association between both polymorphisms and clozapine response.

Calcitonin, used to treat osteoporosis, inhibits bone resorption via receptors located on the osteoclasts. In a study of post-menopausal Caucasian women, Taboulet *et al.* [39] found that women who were heterozygous for a mutation causing proline to leucine change within the calcitonin receptor had a higher bone mineral density at the femoral neck, and a decreased fracture risk, compared to women who were homozygous for either allele. The heterozygote frequency of this polymorphism is much lower in the Japanese population, which could be linked to the higher incidence of vertebral fractures [27].

### Pharmacogenetics of disease pathway genes

Variation in genes, presumably involved in the disease process itself, rather than the absorption or metabolism of the drug, has been found to be associated with variation in drug response. Patients with Alzheimer's disease (AD) are often genotyped for the ApoE4 allele, which is associated both with susceptibility to and poorer prognosis for disease. In a study in the USA, it was found that the E4 allele was also associated with response to the drug tacrine: 80% of patients who did not carry the ApoE4 allele improved after tacrine drug treatment, whereas 60% of patients with the ApoE4 allele actually deteriorated after tacrine treatment [31]. The mechanism of this association is unknown.

In the cardiovascular field, the cholesterol-lowering drug pravastatin was the subject of another pharmacogenetic study. The rarer allele of a polymorphism in the cholesterol esterase transfer protein (CETP) gene was found to be associated with pravastatin response. The B1 allele was associated with higher CETP concentrations, faster progression of atherosclerosis and response to pravastatin, as measured by increase in mean luminal diameter. Patients with the B2B2 genotype (16% of a study group of 807 men) had no significant increase in mean luminal diameter, although their cholesterol levels were still significantly reduced. The B1B2 genotype patients were intermediate in their response to the drug [19].

Although both these studies showed strong associations between genotype and clinical response, neither showed a complete correlation, emphasizing that genetic associations involving genes in the disease pathway, rather than in the target or transporters of the drug, are likely to be complex and multifactorial in nature.

### Toxicogenomics

The concept of using changes in gene expression to predict which compounds are likely to cause toxicity in patients is similar, although in some ways simpler, than using them to predict genes involved in the pathogenesis of disease. The aim of such studies is to create a database of genes whose expression is altered by those compounds which are associated with toxicity in laboratory animals and/

Copyright © 2000 John Wiley & Sons, Ltd.

or human subjects. This database could then be used in early cell-based tests to select out compounds which are likely to have significant toxicity.

Until recently, the only way of studying changes in expression in a several genes simultaneously was using gel-based methods such as differential display. Rodi *et al.* [33] have used the AFLP technology developed by PE GenScope to study liver toxicity. In this technique, mRNA from the cell system of interest is converted into double-stranded cDNA, then cut with specific restriction enzymes and tagged with specific adapters of known sequences. Using this approach, they identified more than 300 genes that differ in expression level by at least two-fold in response to the rodent liver carcinogen phenobarbital. In a pilot study, 10 of these gene fragments were cloned and sequenced, resulting in three clones that matched rat sequences in public databases, five further clones that matched sequences in other species, and two novel sequences.

Gel-based methods remain a very labour-intensive way of monitoring gene expression, so in the past few years microarrays have become the method of choice for this sort of work. Genomic or cDNA clones of interest are amplified by PCR and spotted onto glass or filters using automated systems. The arrays (representing up to 10 000 genes at a time) are then probed with fluorescent-labelled cDNA from test and control systems. Nuwaysir *et al.* [30] have developed a custom cDNA microarray chip, ToxChip v1.0, containing 2090 genes selected for involvement in basic cellular processes as well as responses to toxic insult (see also [10], and **http://www.affymetrix.com/products/Rat_Toxicology.pdf**). However, data from these studies have not yet been published.

### Conclusion

The future of pharmacogenomics with its promise of personalized or tailored medicine, 'the right drug for the right patient', may seem far away. Yet all the building blocks for this future are being assembled and are likely to be put into place over the next 5 years [15,37]. There are many examples in the literature of strong associations between genetic variation and drug response, and some of these even form the basis of accepted clinical tests. For the patient, pharmacogenomics should mean an overall increase in drug efficacy and safety, and this should be the motivation for the development of sound scientific studies to back up the claims of 'tailored' drugs developed by the pharmaceutical industry. For those working in the industry, there is an added motivation in the possibility that pharmacogenomics will be able to reduce size, and therefore cost, of clinical trials. However, there are many issues surrounding the legal, regulatory and ethical framework to these studies that remain unanswered, and a huge amount of education both for the public and healthcare professionals will be needed before the results of this new medicine can be widely accepted [14,32] (see **http://www.shef.ac.uk/uni/projects/sible/sible.html**).

### References

1. Arranz M, Munro J, Owen MJ, *et al.* 1998. Evidence for association between polymorphisms in the promoter and coding regions of the 5–$HT2_A$ receptor gene and response to clozapine. *Mol Psychiatry* **3**: 61–66.
2. Bond C, LaForge KS, Tian M, *et al.* 1998. Single-nucleotide polymorphism in the human μ opioid receptor gene alters β–endorphin binding and activity: possible implications for opiate addiction. *Proc Natl Acad Sci USA* **95**: 9608–9613.
3. Bruss M, Bonisch H, Buhlen M, Nothen MM, Propping P, Gothert M. 1999. Modified ligand binding to the naturally occurring Cys-124 variant of the human serotonin 5-HT1B receptor. *Pharmacogenetics* **9**: 95–102.
4. Büscher R, Herrmann V, Insel PA. 1999. Human adrenoceptor polymorphisms: evolving recognition of clinical importance. *Trends Pharmacol Sci* **20**: 94–99.
5. Carson PEF, Flanagan CL, Ickes CE, Alving AS. 1956. Enzymatic deficiency in primaquine-sensitive erythrocytes. *Science* **124**: 484–485.
6. Collins F, Guyer MS, Charkravarti A. 1997. Variations on a theme: cataloging human DNA sequence variation. *Science* **278**: 1580–1158.
7. Drazen J, Yandava CN, Dube L, *et al.* 1999. Pharmacogenetic association between ALOX5 promoter genotype and the response to anti–asthma treatment. *Nature Genet* **22**: 168–170.
8. Evans DAP, Manley KA, McKusick VA. 1960. Genetic control of isoniazid metabolism in man. *Br Med J* **2**: 485–491.
9. Evans W, Relling MV. 1999. Pharmacogenomics: Translating Functional Genomics into Rational Therapeutics. *Science* **286**: 487
10. Farr S, Dunn RT. 1999. 2nd Concise review: gene expression applied to toxicology. *Toxicol Sci* **50**: 1–9.
11. Gonzalez F, Skoda RC, Kimura S, *et al.* 1988. Characterization of the common genetic defect in humans deficient in debrisoquine metabolism. *Nature* **331**: 442–426.
12. Haas M, Yilmaz N, Schmidt A, *et al.* 1998. Angiotensin-converting enzyme gene polymorphism determines the antiproteinuric and systemic hemodynamic effect of enalapril in patients with proteinuric renal disease. Austrian Study

Copyright © 2000 John Wiley & Sons, Ltd. 

Group of the Effects of Enalapril Treatment in Proteinuric Renal Disease. *Kidney Blood Press Res* **21**: 66–69.
13. Hodgson J. 1999. Analysts., firms pour cold water on SNP Consortium. *Nature Biotechnol* **17**: 526
14. Hodgson J, Marshall A. 1998. Pharmacogenomics: will the regulators approve? *Nature Biotechnol* **16**: 243–246.
15. Housman D, Ledley FD. 1998. Why pharmacogenomics? Why now? *Nat Biotechnol* **16**: 492–493.
16. Hughes HB, Biehl JP, *et al*. 1954. Metabolism of isoniazid in man as related to the occurrence of peripheral neuritis. *Am Rev Tuberc* **70**: 266–273.
17. Kalow W. 1956. Familial incidence of low pseudocholinesterase level. *Lancet* **2**: 576–577.
18. Kleyn P, Vesell ES. 1998. Genetic variation as a guide to drug development. *Science* **281**: 1820–1821.
19. Kuivenhoven J, Jukema JW, Zwinderman AH, *et al*. 1998. The role of a common variant of the cholesteryl ester transfer protein gene in the progression of coronary atherosclerosis. The Regression Growth Evaluation Statin Study Group. *N Engl J Med* **338**: 86–93.
20. Lazarou J, Pomeranz BH, Corey PN. 1998. Incidence of adverse drug reactions in hospitalized patients: a meta-analysis of prospective studies. *JAMA* **279**: 1200–1205.
21. Lennard LL, Lilleyman JS, Van Loon J, Weinshilboum RM. 1990. Genetic variation in response to 6–mercaptopurine for childhood acute lymphoblastic leukaemia. *Lancet* **336**: 225–229.
22. Mahgoub A, Idle JR. , Dring LG, Lancaster R, Smith RL. 1977. Polymorphic hydroxylation of debrisoquine in man. *Lancet* **2**: 584–586.
23. Marshall A. 1998. Laying the foundations for personalized medicines. *Nature Biotechnol* **16**: 954–957.
24. Marshall A. 1998. Getting the right drug into the right patient. *Nature Biotechnol* **15**: 1249–1252.
25. Masood E. 1999. As consortium plans free SNP map of human genome. *Nature* **398**: 545–546.
26. Meyer U, Zanger UM. 1997. Molecular mechanisms of genetic polymorphisms of drug metabolism. *Ann Rev Pharmacol Toxicol* **37**: 269–296.
27. Nakamura M, Zhang ZQ, Shan L, *et al*. 1997. Allelic variants of human calcitonin receptor in the Japanese population. *Hum Genet* **99**: 38–41.
28. Nebert DW. 1997. Polymorphisms in drug–metabolizing enzymes: what is their clinical relevance and why do they exist?. *Am J Hum Genet* **60**: 265
29. Nebert D, Ingelman-Sundberg M, Daly AK. 1999. Genetic epidemiology of environmental toxicity and cancer susceptibility: human allelic polymorphisms in drug-metabolizing enzyme genes, their functional importance, and nomenclature issues. *Drug Metab Rev* **31**: 467–487.
30. Nuwaysir E, Bittner M, Trent J, Barrett JC, Afshari CA. 1999. Microarrays and toxicology: the advent of toxicogenomics. *Mol Carcinog* **24**: 153–159.
31. Poirier J, Delisle MC, Quirion R, *et al*. 1995. Apolipoprotein E4 allele as a predictor of cholinergic deficits and treatment outcome in Alzheimer disease. *Proc Natl Acad Sci U S A* **92**: 12260–12264.
32. Pharmacogenomics Supplement. 1998. *Nature Biotechnol.* **16**(10).
33. Rodi C, Bunch RT, Curtiss SW, *et al*. 1999. Revolution through genomics in investigative and discovery toxicology. *Toxicol Pathol* **27**: 107–110.
34. Sadee W. 1999. Pharmacogenomics. *Br Med J* **319**: 1286
35. Silverman E, In KH, Yandava C, Drazen JM. 1998. Pharmacogenetics of the 5–lipoxygenase pathway in asthma. *Clin Exp Allergy* **28** (suppl 5): 164–170.
36. Smeraldi E, Zanardi R, Benedetti F, Di Bella D, Perez J, Catalano M. 1998. Polymorphism within the promoter of the serotonin transporter gene and antidepressant efficacy of fluvoxamine. *Mol Psychiat* **3**: 508–511.
37. Schmidt K. 1998. Just for you. *New Scientist 14 November.*
38. Spears B. 1999. Applying the science: we're only a step away. Presented at Symposium on Genetics in the Pharmaceutical Industry, San Francisco, CA.
39. Taboulet J, Frenkian M, Frendo JL, Feingold N, Jullienne A, de Vernejoul MC. 1998. Calcitonin receptor polymorphism is associated with a decreased fracture risk in post–menopausal women. *Hum Mol Genet* **7**: 2129–2133.
40. Weber WW. 1997. *Pharmacogenetics*. Oxford University Press: Oxford.
41. Weinshilboum R, Sladek SL. 1980. Mercaptopurine pharmacogenetics: monogenic inheritance of erythrocyte thiopurine methyltransferase activity. *Am J Hum Genet* **32**: 651–662.

Copyright © 2000 John Wiley & Sons, Ltd. 
